# X-ray interference fringes from a weakly bent plane-parallel crystal with negative strain gradient

**DOI:** 10.1107/S2053273319011859

**Published:** 2019-10-07

**Authors:** Tomoe Fukamachi, Sukswat Jongsukswat, Dongying Ju, Riichirou Negishi, Keiichi Hirano, Takaaki Kawamura

**Affiliations:** a Saitama Institute of Technology, Fukaya, Saitama 369-0293, Japan; b COBEXM Co., Ltd, Ota, Gunma 373-8567, Japan; c Institute of Material Structure Science, KEK-PF, High Energy Accelerator Research Organization, Tsukuba, Ibaraki 305-0801, Japan; d Center for Ceramic Matrix Composites, Tokyo University of Technology, Hachioji, Tokyo 192-0982, Japan

**Keywords:** interference fringes, mirage fringes, X-ray beam trajectory, bent crystal, multiple Bragg diffraction, dynamical theory of X-ray diffraction

## Abstract

Under the anomalous transmission condition in the Bragg mode, X-ray interference fringes were observed between two beams with different hyperbolic trajectories in a very weakly bent plane-parallel perfect crystal with negative strain gradient. The origin of the fringes was analysed based on the dynamical theory of diffraction for a distorted crystal. In the reflected beam from the entrance surface, the interference fringes were observed between once- and twice-reflected beams from the back surface. In the transmitted beam from the back surface, the interference fringes were observed between the direct beam and once-reflected beam from the entrance surface. In the emitted beam from the lateral surface, the interference fringes were observed between the beams after different numbers of reflections in the crystal. The multiply reflected beams were formed by a combined result of long propagation length along the beam direction with large divergence of the refracted beams when the strain gradient was negative. The period of these interference fringes was sensitive to very weak strain, of the order of 10^−7^.

## Abstract 2

In the waves emitted from the entrance, back and lateral surfaces of a very weakly bent plane-parallel perfect crystal with negative strain gradient, X-ray interference fringes between two refracted beams with different hyperbolic trajectories were observed when the strain was very weak, of the order of 10^−7^.

## 1. Introduction   

**Figure 1:**
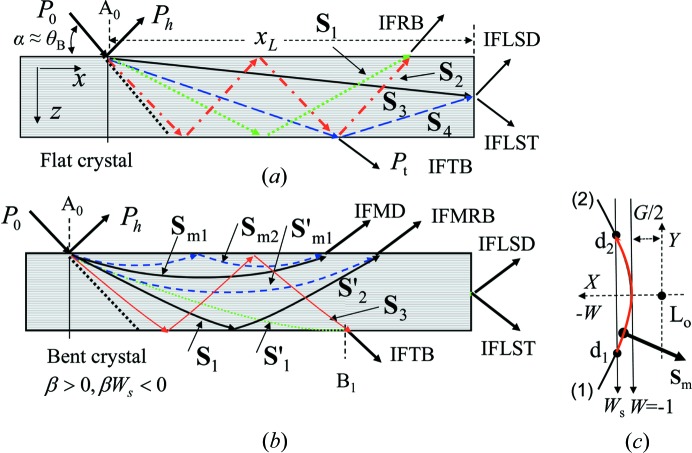
Schematic illustrations of the beam trajectories in a plane-parallel crystal. (*a*) Beam trajectories in an unbent crystal. *P*_0_ represents the incident wave on the surface, *P*_h_ the diffracted wave and *P*_t_ the transmitted wave. (*b*) Beam trajectories in a bent crystal when the X-ray is incident on the expanded surface due to bending (β > 0). **S**′_1_ represents the beam touching the back surface at B_1_ and **S**_3_ the beam reflected from the entrance surface after reflection from the back surface. (*c*) The variation range of the tie point on the dispersion surface for mirage beams. *d*_1_ on branch (1) is the tie point at the incident point and *d*_2_ on branch (2) is that at the emitted point. L_o_ is the Lorentz point and *G* the Bragg gap. The distance from the incident point of the X-ray (A_0_) to the edge of the crystal is 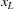.

When X-rays are incident on a thin plane-parallel perfect crystal in the symmetric Bragg geometry, the refracted beam in the crystal reaches the back surface as illustrated in Fig. 1 ▸(*a*). A part of the beam is reflected (**S**_4_) and the rest is emitted from the back surface as the transmitted beam (*P*_t_). The refracted beam is referred to as the beam corresponding to the Poynting vector excited at a point on the dispersion surface defined in the dynamical theory of diffraction. The refracted beam is called the wavefield by Authier (2001 ▸). In the two-wave approximation, it is composed of two waves: one is propagating in the forward direction and the other in the diffracted-wave direction. In an anomalous transmission condition, the divergence of the refracted beam is much larger than that of the incident wave (Authier, 2001 ▸). If the divergence angle of the refracted beam is large enough, interference fringes can be formed between once (**S**_1_)- and twice (**S**_2_)-reflected beams from the back surface (abbreviated as IFRB) as shown in Fig. 1 ▸(*a*). Similar interference fringes can be observed in the transmitted beam (IFTB) from the back surface. If the crystal is small along the incident azimuth direction (*x*) compared with the propagation length of the X-ray, interference fringes can be observed in the emitted beams from the lateral surface both in the diffracted- (IFLSD) and the transmitted-wave directions (IFLST). Both IFLSD and IFLST have been observed by Fukamachi *et al.* (2004 ▸, 2005 ▸) from a thin Ge plane-parallel crystal. These fringes were formed by interference between the beams directly propagating to the lateral surface (**S**_3_) and once reflected from the back surface (**S**_4_) (Hirano *et al.*, 2008 ▸, 2009*a* ▸,*b* ▸; Fukamachi, Hirano *et al.*, 2011 ▸).

When a perfect crystal is bent with positive strain gradient (strain gradient parameter defined below β > 0), the trajectory of the refracted beam shows a hyperbolic form opening up and some refracted beams return to the entrance surface without reaching the back surface such as **S**_m1_, **S**_m2_ and **S**′_m1_ in Fig. 1 ▸(*b*), as pointed out by Gronkowski & Malgrange (1984 ▸). These refracted beams are called mirage beams by Authier (2001 ▸). Fig. 1 ▸(*c*) shows the tie points corresponding to mirage beams in the range from *d*_1_ to *d*_2_ on the dispersion surface. The interference fringes formed by two mirage diffraction beams (IFMD) have been observed by Fukamachi *et al.* (2010 ▸) and Jongsukswat *et al.* (2012*a* ▸, 2013 ▸). It was pointed out by Fukamachi, Jongsukswat *et al.* (2011 ▸) and Jongsukswat *et al.* (2012*b* ▸) that interference fringes between the mirage diffraction beam **S**′_m1_ and the beam reflected from the back surface **S**′_2_ (IFMRB) are expected to be observed as shown in Fig. 1 ▸(*b*).

**Figure 2:**
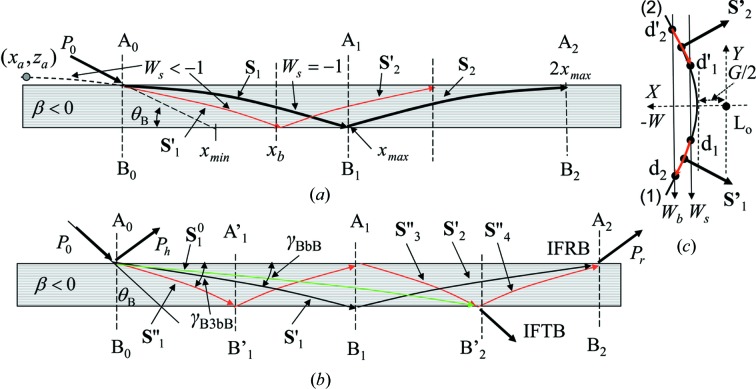
Schematic illustrations of the beam trajectories in a bent plane-parallel crystal for β < 0. (*a*) **S**_1_ represents the beam excited when *W*_s_ = −1. It propagates the longest distance (*x*_max_) in the *x* direction, reaches the back surface at B_1_ and is partly reflected as the beam **S**_2_. **S**′_1_ represents a beam excited when *W*_s_ < −1 and **S**′_2_ the corresponding reflected beam from the back surface. (*b*) Beam trajectories to form IFRB and IFTB. IFRB from A_2_ are formed by interference between the beam **S**′_2_ once reflected and the beam **S**′′_4_ twice reflected from the back surface. IFTB from B′_2_ are formed by interference between the beam **S** ^0^_1_ reaching directly the back surface and the beam **S**′′_3_ once reflected from the back surface. (*c*) The variation range of the tie point on the dispersion surface for **S**′_1_ and **S**′_2_. The range from *d*_1_ to *d*_2_ on branch (1) is for **S**′_1_ and that from *d*′_2_ to *d*′_1_ on branch (2) is for **S**′_2_. 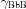 and 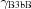 are the angles of the refracted beams in the BbB and the B3bB modes from the surface, respectively.

If a perfect crystal is bent with negative strain gradient (β < 0), the trajectory of the refracted beam shows a hyperbolic form opening down and the beam always reaches the back surface as shown in Fig. 2 ▸(*a*). Fig. 2 ▸(*c*) shows that the tie points corresponding to the refracted beam **S**′_1_ and **S**′_2_ move on the dispersion surface in the range indicated by red lines. IFRB can be observed between two beams, *i.e.* one (**S**′_2_) reflected once from the back surface and the other (**S**′′_4_) reflected twice from the back surface in Fig. 2 ▸(*b*). γ is the angle of the refracted beam from the surface at the incident point. Similar interference fringes can be observed between two beams *n* and *n* + 1 (*n* is a positive integer) times reflected from the back surface. IFTB also can be observed for β < 0.

In this paper, we will report on the measurement of IFRB, IFTB, IFLSD and IFLST from a weakly bent Si plane-parallel crystal for β < 0 and the comparison of these interference fringes with those for β > 0. We will analyse the interference fringes of IFRB and IFTB based on the dynamical theory of diffraction for a distorted crystal and reveal some characteristics of them for β < 0.

## 2. Experimental   

**Figure 3:**
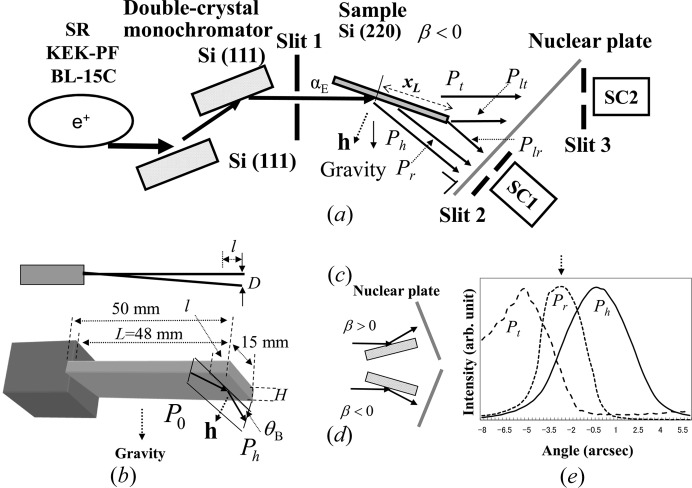
(*a*) A schematic diagram of the measuring system. *x_L_* is the distance between the incident point of the X-rays and the crystal edge of the entrance surface. SC1 and SC2 are the scintillation counters set up in the reflected- and transmitted-wave directions, respectively. (*b*) Sample geometries. (*c*) and (*d*) the incident- and reflected-wave geometries with respect to the samples for β > 0 and β < 0, respectively. (*e*) The measured rocking curves of *P*_h_, *P*_t_ and *P*_r_.

The sample was a plane-parallel single Si crystal. The top (entrance) and bottom (back) surfaces of the crystal were polished by a non-disturbance polishing method at Sharan Inc. The size was 50 mm long, 15 mm wide and 0.28 mm thick. One end of the sample was clamped and the other end was free along the gravity direction as shown in Fig. 3 ▸(*b*). The sample was bent due to gravity and the residual strain. The experiments were carried out using X-rays from synchrotron radiation at the bending-magnet beamline 15C, Photon Factory, Tsukuba, Japan. The measuring optical system is shown in Fig. 3 ▸(*a*). The X-rays were σ-polarized and had a very narrow band of energy achieved by using an Si(111) double-crystal monochromator. The X-ray energy was 11 100 eV, which was determined by measuring XANES (X-ray absorption near-edge structure) from a thin Ge plate near the Ge *K* absorption edge (11 103 eV) with an accuracy of ±0.5 eV. The distance from the source to slit 1 was 30 m and that from slit 1 to the sample was 300 mm. The vertical width of slit 1 was 0.02 mm. In Fig. 3 ▸(*a*), *P*_h_, *P*_r_, *P*_t_, *P*_lr_ and *P*_lt_ are the intensities of the diffracted wave, the reflected beam except for the diffracted beam, the transmitted beam, the emitted beam from the lateral surface in the diffracted-wave direction and that in the transmitted-wave direction, respectively. As shown in Fig. 3 ▸(*b*), the X-rays were incident on the crystal with the azimuth perpendicular to the bending direction. The incident glancing angle was fixed at the angle where the rocking curve of *P*_r_ in Fig. 3 ▸(*e*) showed the peak and the anomalous transmission was maximized. The X-ray intensities were measured by the scintillation counters (SC1 and SC2) and recorded on a nuclear plate (ILFORD L4; emulsion thickness, 25 µm). The incident and reflected X-ray geometries with respect to the sample are shown for positive and negative values of β in Figs. 3 ▸(*c*) and 3 ▸(*d*), respectively. For observation of IFRB, it is important to make the value of |β| small. If *l* denotes the distance from the free edge to the incident plane and *L* that from the free edge to the fixed edge, |β| is proportional to 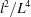 (Jongsukswat *et al.*, 2013 ▸). In the present experiment the distance *l* was between 3.25 and 4 mm, and *L* was 48 mm.

**Figure 4:**
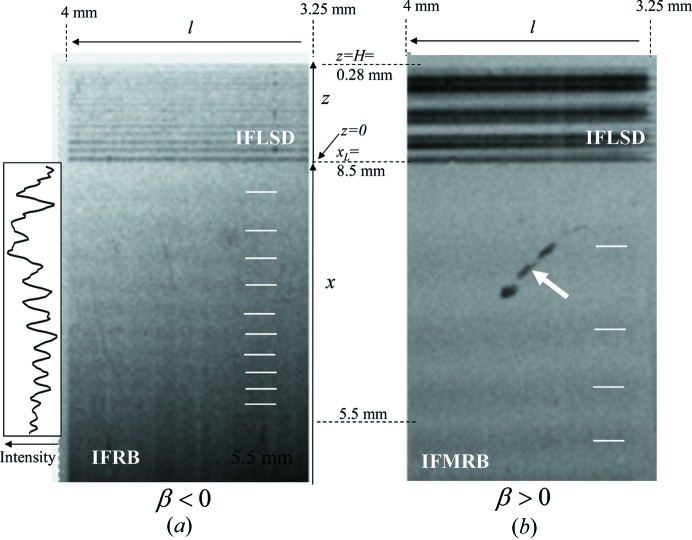
Section topographs of Si(220) in the Bragg mode recorded in the diffracted-wave direction for (*a*) β < 0 and (*b*) β > 0. The distance *x_L_* is 8.5 mm. The left side of (*a*) shows the digitized intensities of IFRB. The horizontal direction is the distance *l*.

**Figure 5:**
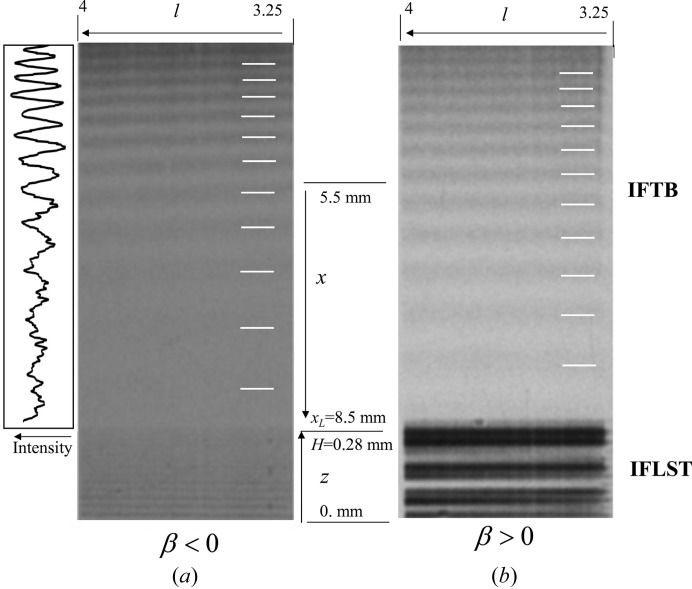
Section topographs of Si(220) in the Bragg mode recorded in the transmitted-wave direction for (*a*) β < 0 and (*b*) β > 0. The distance *x_L_* is 8.5 mm. The left side of (*a*) shows the digitized intensities of IFTB.

Fig. 4 ▸ shows the section topographs of Si(220) measured in the diffracted direction under only the gravity force. Fig. 5 ▸ shows those measured in the transmitted-wave direction. The strain gradient was negative in Fig. 4 ▸(*a*) and Fig. 5 ▸(*a*), and positive in Fig. 4 ▸(*b*) and Fig. 5 ▸(*b*). The exposure time for taking one topograph was 120 min for Fig. 4 ▸(*a*), 60 min for Fig. 5 ▸(*a*), 15 min for Fig. 4 ▸(*b*) and 30 min for Fig. 5 ▸(*b*). In Fig. 4 ▸(*a*) the interference fringes in the upper part are attributed to IFLSD and those in the lower part to IFRB. The vertical direction of the lower part corresponds to the distance *x* from the incident point of the X-rays on the entrance surface and that in the upper part corresponds to the distance *z* from the edge of the entrance surface on the lateral surface. The horizontal direction corresponds to distance *l*. Ten dark lines are observed in IFRB as indicated by white lines. The plot of digitized intensities of IFRB is given on the left side of the figure, showing ten peaks in the range of *x* between 5.5 and 8.5 mm. The number of interference fringes in Fig. 4 ▸(*a*) is larger than that in Fig. 4 ▸(*b*) where the number is three in the same range of *x*. The distance between two adjacent dark lines in IFRB increases as a function of *x*, which is a similar variation to that in IFMRB shown in Fig. 4 ▸(*b*). The distance between two adjacent dark lines in IFLSD in Fig. 4 ▸(*a*) is smaller than that in Fig. 4 ▸(*b*). The dark contrast indicated by the white arrow in Fig. 4 ▸(*b*) is accretions on the nuclear emulsion plate. In Fig. 5 ▸(*a*) the interference fringes in the upper part are attributed to IFTB and those in the lower part to IFLST. The number of dark lines in IFTB indicated by white lines is five in the same range of *x*. The horizontal direction is the same as in Fig. 4 ▸. The distance between two adjacent dark lines increases as a function of *x*, which is a similar variation to that in IFRB. In Fig. 5 ▸(*b*) the interference fringes in the upper part are attributed to IFTB and those in the lower part to IFLST. The number of dark lines in ITFB for β < 0 is almost the same as that for β > 0 in the same range of *x*.

## 3. Theoretical basis   

### 3.1. Beam trajectory   

In the dynamical theory of diffraction, the deviation parameter *W* from an exact Bragg condition is defined by 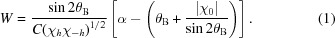
Here α is the incident glancing angle, 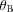 is the Bragg angle, *C* the polarization factor and 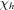 the **h**th Fourier component of X-ray polarizability. By using the deviation parameter at the incident point (*W*_s_), the trajectory of the refracted beam for 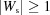 in a non-absorbing bent crystal with constant strain gradient is given by 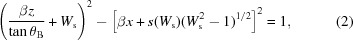
according to Gronkowski & Malgrange (1984 ▸). The coordinates *x* and *z* are parallel and inward normal to the crystal surface and the origin is taken at the incident point of the X-ray. 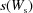 is equal to 1 for 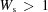 and −1 for 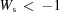. The strain gradient parameter β is given by
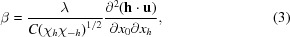
where **h** is the reciprocal-lattice vector, **u** the displacement vector of an atom and λ the X-ray wavelength. 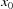 and 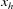 are the coordinates of the transmitted- and the diffracted-wave directions, respectively. According to Yan & Noyan (2006 ▸) and Yan *et al.* (2007 ▸), β is related to the strain gradient 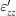 as 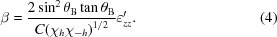
The strain in a bent plane-parallel crystal of thickness *H* is given by 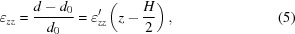
where *d* and *d*_0_ are the distances between two adjacent lattice planes normal to the crystal surface with and without distortion due to bending, respectively.

The trajectory of the refracted beam becomes a hyperbola for 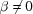 by using equation (2). Its vertex position (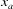, 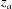) is given by 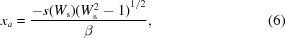


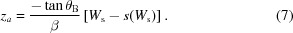
The beam trajectory for positive strain gradient has been studied in previous works (Fukamachi *et al.*, 2010 ▸; Fukamachi, Jongsukswat *et al.*, 2011 ▸; Jongsukswat *et al.*, 2012*a* ▸,*b* ▸, 2013 ▸). The vertex of the hyperbola is located inside the crystal when β > 0 and 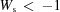 according to equation (7). Some refracted beams such as **S**_m1_ and **S**_m2_ return to the entrance surface without touching the back surface as depicted in Fig. 1 ▸(*b*). On the other hand, when the strain gradient is negative (β < 0), the vertex is located outside the entrance surface. The beam trajectory is bent downwards in the crystal and all the beams reach the back surface as depicted in Figs. 2 ▸(*a*) and 2 ▸(*b*). The refracted beam corresponding to 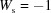 propagates in the crystal when 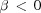 , while such a refracted beam is not excited when β > 0. Using equation (2), the value of the deviation parameter 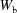 at the back surface is given by 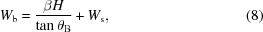
which satisfies the relation 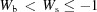. The *x* component of the propagation length of the X-ray from the incident point to the back surface is given as 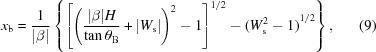
by setting *z* = *H* in equation (2). The distance 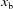 decreases monotonically as a function of 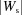 for 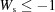.

The maximum value of *x* for observing IFTB is determined by the exit point of the refracted beam for γ = 0 (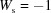). The value 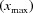 is half the maximum value of *x* for observing IFRB (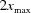) for γ = 0 as depicted in Fig. 2 ▸(*a*). Using equation (9), the value of 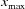 is 9.6, 7.9 and 6.8 mm when 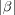 is 0.02, 0.03 and 0.04 mm^−1^, respectively. In the experiment, IFTB is observed up to 8 mm as shown in Fig. 5 ▸(*a*). 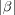 must be less than 0.03 mm^−1^. The uppermost observed point 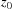 of IFLSD is given by the exit point of the beam for γ = 0 from the lateral surface. The point 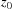 is 0.21, 0.11 and 0.055 mm when 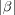 is 0.02, 0.01 and 0.005 mm^−1^, respectively. As the measured value of 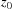 is around 0.14 mm, 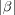 must be more than 0.01 mm^−1^. Then it is reasonable to take the value of 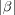 as 0.02 mm^−1^ within the error of ±0.01 mm^−1^. To determine the value of β from only IFRB, it is necessary to measure the interference fringes for *x* more than 17 mm as will be shown later.

### 3.2. Interference fringes between two reflected beams from the back surface   

In Fig. 2 ▸(*b*), as IFRB emitted from A_2_ are observed in the range of *x* from around 5.5 to 8.5 mm, they are attributed to the interference fringes between the once-reflected beam (**S**′_2_) and the twice-reflected beam (**S**′′_4_) from the back surface. The beam once reflected from the back surface is called the beam in the BbB mode hereafter, since it is incident on the entrance surface (A_0_) satisfying the boundary condition of the Bragg mode, reflected from the back surface (B_1_) satisfying that of the Bragg mode and emitted from the entrance surface (A_2_) satisfying that of the Bragg mode. It is possible for these beams to interfere with the beam in the B5bB mode. But the amplitude of the beams in the B5bB mode is much smaller than that in the BbB mode, because the amplitude becomes small after each reflection from the entrance or the back surface. In the following, the contribution of the beam in the B5bB mode is ignored.

The amplitude of the beam in the BbB mode is denoted as *E*_BbB_. Similarly, the amplitude corresponding to the beam twice reflected from the back surface, *i.e.* the beam in the B3bB mode, is denoted as *E*_B3bB_. Then the amplitudes of *E*_BbB_ and *E*_B3bB_ from a non-absorbing crystal can be written as 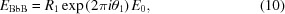


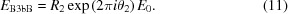
Here 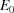 is the amplitude of the incident X-ray. 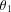 and 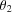 are the phase factors of 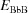 and 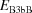, respectively. The phase factor 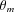 (*m* = 1 and 2) is given by 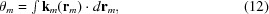
with 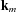 being the wavevector and 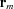 the position vector. 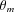 can be divided into two parts as 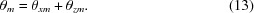


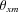
 and 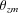 are obtained from the *x* and *z* components of 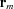. The details of the calculation of equation (13) were given by Fukamachi *et al.* (2010 ▸) and Jongsukswat *et al.* (2012*b* ▸). In equations (10) and (11), 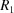 and 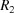 are given by 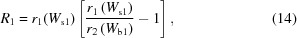


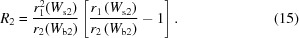
The reflection coefficients are given as 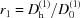 and 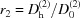, with 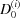 and 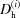 being the amplitudes of electric displacement of the *i*th branch of the transmitted and diffracted waves, respectively. *W*_s1_ and *W*_s2_ are the deviation parameters for the beams **S**_1_′ and **S**_1_′′ at the entrance surface, and *W*_b1_ and *W*_b2_ are those at the back surface. The intensity of IFRB from A_2_ in Fig. 2 ▸(*b*) is given by 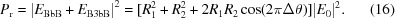
The phase factor Δθ is given by 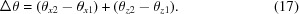



## 4. Discussion   

### 4.1. Comparison between measured and calculated interference fringes   

**Figure 6:**
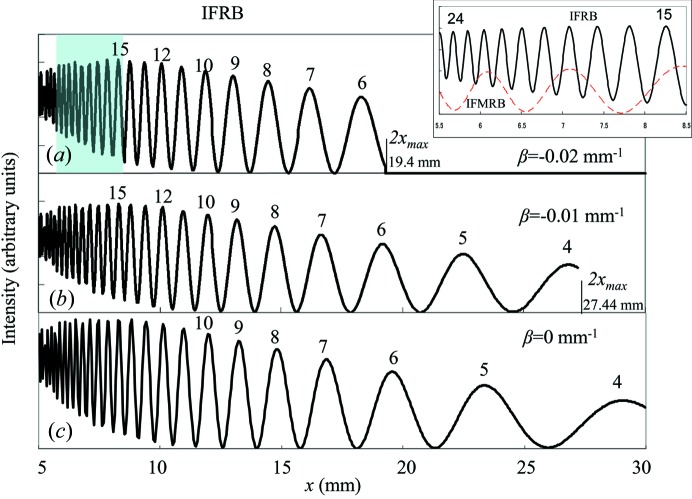
Calculated intensities of IFRB in the range of *x* from 5 to 30 mm for β being (*a*) −0.02 mm^−1^, (*b*) −0.01 mm^−1^ and (*c*) 0.0 mm^−1^. The inset shows the calculated intensities of IFRB (black curve) for β = −0.02 mm^−1^ and IFMRB for β = 0.02 mm^−1^ (red dashed curve) in the range of *x* from 5.5 to 8.5 mm [the shaded region in (*a*)].

Intensities of IFRB calculated using equation (16) are shown in Figs. 6 ▸(*a*), 6 ▸(*b*), 6 ▸(*c*) for β = −0.02, −0.01 and 0.0 mm^−1^, respectively. The integer value at each peak denotes the value of Δθ in equation (17). The distance between two adjacent peaks increases as the distance *x* increases or the value of 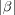 decreases. The inset of Fig. 6 ▸ shows the variations of IFRB (the solid curve) for β = −0.02 mm^−1^ in the range of *x* between 5.5 and 8.5 mm, which corresponds to the shaded area in Fig. 6 ▸(*a*). For comparison, the calculated intensity variation of IFMRB is also shown for β = 0.02 mm^−1^. The IFMRB intensities are calculated by assuming the interference between the mirage diffraction beam **S**′_m1_ and the beam **S**′_2_ reflected from the back surface in Fig. 1 ▸(*b*) and using the formula given by Jongsukswat *et al.* (2012*b* ▸). The number of interference fringes of IFRB is ten and that of IFMRB is three. The distance between two adjacent peaks increases as the distance *x* increases in both the IFRB and IFMRB. The calculated results in Fig. 6 ▸(*a*) and the inset agree quite well with the observed results in Fig. 4 ▸ at least qualitatively. Based on this good agreement the strain gradient parameter of the present crystal is determined to be β = −0.02 mm^−1^. In order to determine the value of β only from IFRB, it is necessary to measure the interference fringes for *x* more than 17 mm as the difference in the period of the fringes becomes conspicuous for large *x* as can be seen in Fig. 6 ▸. It is possible for the beams **S**′_m1_ and **S**′_2_ (in the BbB mode) to interfere with the beam in the B3bB mode to form the IFMRB. However, as described in Section 3.2, the amplitude of the beam in the B3bB mode is much smaller than that of the mirage diffraction beam **S**′_m1_ because of three more reflections than **S**′_m1_.

**Figure 7:**
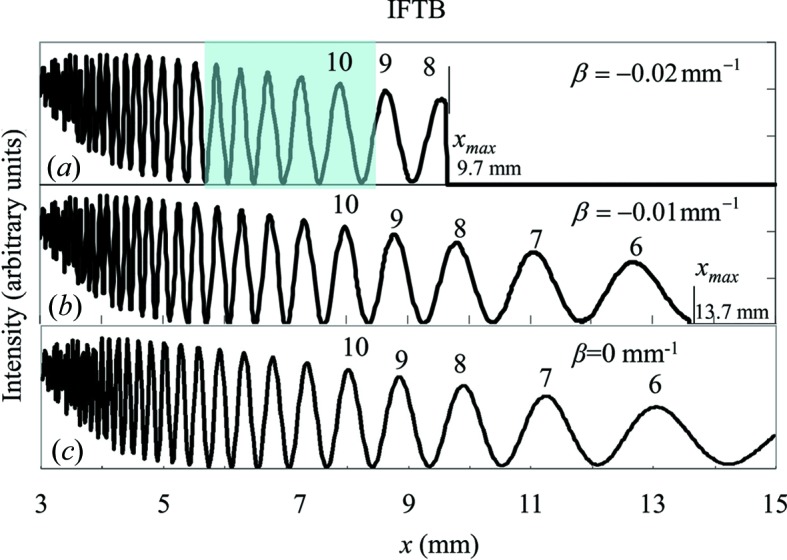
Calculated intensities of IFTB in the range of *x* from 3 mm to *x*_max_ for β being (*a*) −0.02 mm^−1^, (*b*) −0.01 mm^−1^ and (*c*) 0.0 mm^−1^.

**Figure 8:**
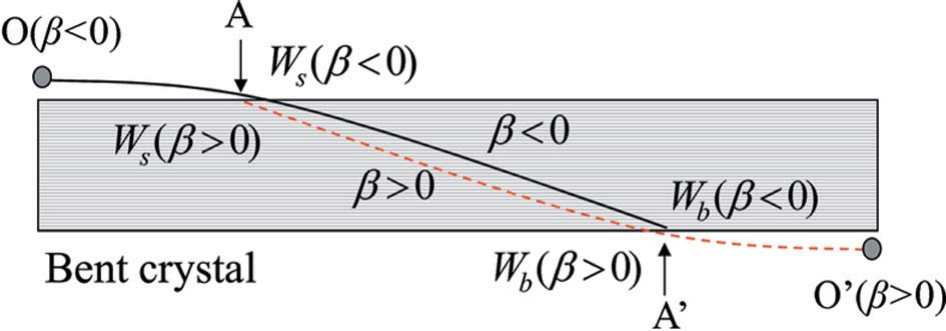
Beam trajectories in a bent plane-parallel crystal for the same 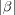. The black solid curve represents the beam trajectory for β < 0 and the red dashed curve that for β > 0. O is the vertex of the hyperbola for β < 0 and O′ that for β > 0.

The calculated intensities of IFTB are shown in Figs. 7 ▸(*a*), 7 ▸(*b*), 7 ▸(*c*) for β = −0.02, −0.01 and 0 mm^−1^, respectively. The distance between two adjacent peaks increases as *x* increases or 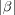 decreases. There are five peaks in the shaded range of *x* between 5.5 and 8.5 mm in Fig. 7 ▸(*a*), which agrees with the experimental result that there are five dark lines in Fig. 5 ▸(*a*) in the same range. The distance between two adjacent peaks in IFTB for β < 0 in Fig. 5 ▸(*a*) is nearly the same as that for β > 0 in Fig. 5 ▸(*b*) when 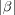 is the same. Beam trajectories are schematically shown for β < 0 (the black solid curve) and for β > 0 (the red dashed curve) for the same 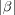 in Fig. 8 ▸. The relations 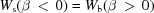 and 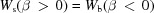 hold and the path lengths of the two trajectories [from A to A′ for 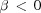 (black solid curve) and A′ to A for 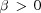 (red dashed curve)] are the same. The values of Δθ are the same for these two trajectories. The number of oscillations of IFTB for 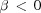 should be the same as that for 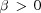 when 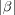 is the same.

**Figure 9:**
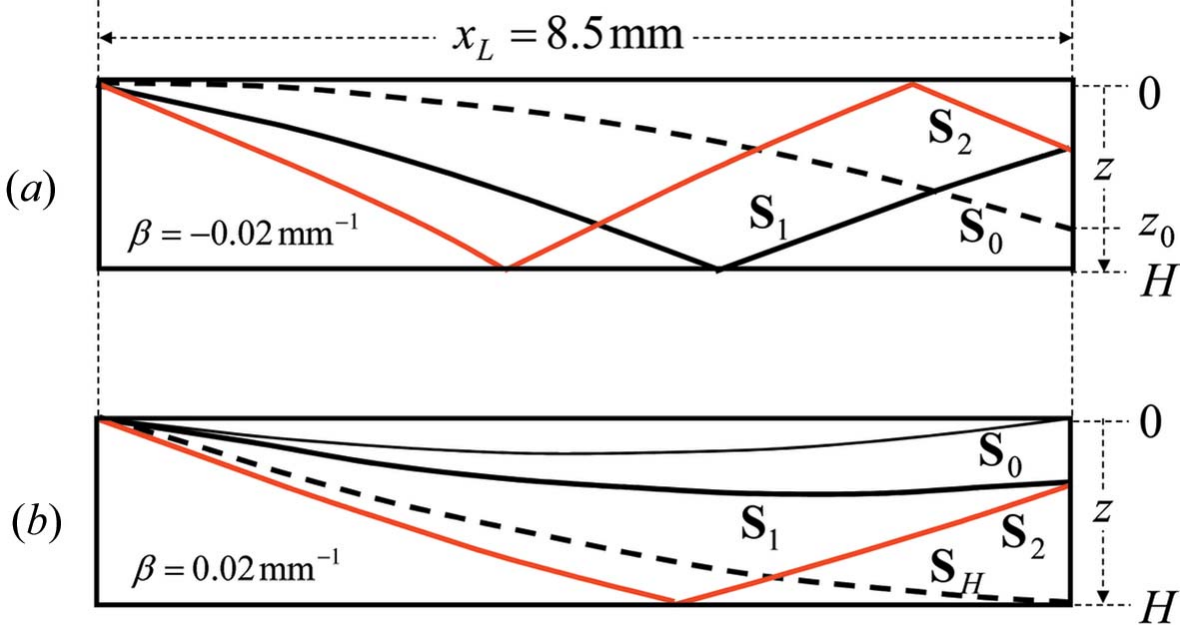
The calculated trajectories of the beams to form IFLSD and IFLST for (*a*) β = −0.02 mm^−1^ and (*b*) β = 0.02 mm^−1^. *x_L_* = 8.5 mm and *H* = 0.28 mm.

In Fig. 4 ▸ IFLSD are also shown both for β < 0 (*a*) and β > 0 (*b*). IFLSD for β < 0 are more clearly observed for small *z* than for large *z* in the upper part of Fig. 4 ▸(*a*). The spacing of the two adjacent dark lines is approximately 35 µm. When β > 0, four dark bands are observed between *z* = 0 and *H* as shown in the upper part of Fig. 4 ▸(*b*). The spacing of the two adjacent bands is approximately 70 µm, which is twice larger than that in Fig. 4 ▸(*a*). The value of β in Fig. 4 ▸(*a*) is −0.02 mm^−1^ and the corresponding beam trajectories are depicted in Fig. 9 ▸(*a*). The beam **S**_0_ (the broken curve) corresponding to 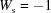 reaches the lateral surface directly at 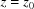 (≃ 0.21 mm). No beam in the BL mode comes to the region between 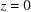 and 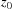 on the lateral surface. The notation L of BL mode denotes that the beam emitted from the lateral surface satisfies the boundary condition of the Laue mode. The observed IFLSD should be formed by interference between the beam **S**_1_ in the BbL mode (black solid curve) and the beam **S**_2_ in the B2bL mode (red solid curve). The beam trajectories for β = 0.02 mm^−1^ are depicted in Fig. 9 ▸(*b*). The beam **S**_0_ (thin solid curve) reaches the point (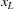, 0) and the beam **S** *_H_* (the broken curve) reaches the point (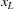, *H*). Here, 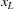 is the distance from the incident point to the edge of the crystal surface. If the parameters 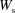 for **S**_0_ and **S** *_H_* are denoted as 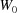 and 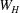, respectively, the beam **S**_1_ is in the BL mode when the parameter 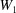 (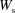 for **S**_1_) satisfies the condition 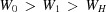, and the beam **S**_2_ is in the BbL mode when the parameter 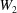 (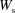 for **S**_2_) satisfies 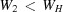. The IFLSD in Fig. 4 ▸(*b*) is attributed to the interference between the beams in the BL mode (black solid curve) and in the BbL mode (red solid curve).

When 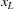 is larger than the crystal thickness (*H*), the spacing of two adjacent peaks of IFLSD between the beams in the BL and the BbL modes 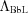 is given by 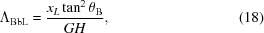
for β = 0. Here *G* is the Bragg gap given by *G* = 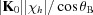 with 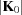 being the wavenumber of the X-ray. Similarly, the spacing of IFLSD between the beams in the BbL and the B2bL modes 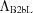 is given by 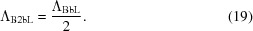
By using equations (18) and (19), 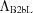 is calculated to be 31 µm, which agrees quite well with the measured value of 35 µm. The crystal was actually bent and 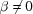 in the present experiment. But the obtained value of 31 µm cannot be much different from the actual value, because equation (19) is a good approximation when 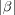 is very small and the trajectories 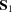 and 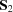 are almost straight.

By using equations (4) and (5), the values of strain gradient and strain on the crystal surface (*z* = 0) are estimated to be 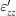 = 1.9 × 10^−6^ mm^−1^ and 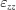 = −2.7 × 10^−7^, when the value of 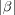 is 0.02 mm^−1^. This value is consistent with the previous result by Jongsukswat *et al.* (2012*b* ▸). If we assume that the strain is only due to gravity, the value of 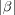 at the distance *l* between 3.25 and 4 mm is 9.35 × 10^−4^ mm^−1^ using the Young’s modulus (105–185 GPa) and Poisson’s ratio (0.17–0.33) of Si. This is approximately 5% of the measured value. On the other hand, the value of 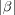 due to the residual strain by Jongsukswat *et al.* (2013 ▸) is 82.3 × 10^−4^ mm^−1^, which is approximately half but close to the current measured value. By considering that residual strain varies depending on the shape, processing, use history and so on, the measured strain should be attributed to mostly residual strain and partly (5%) to strain due to gravity.

### 4.2. Angular amplification of IFRB for β < 0   

**Figure 10:**
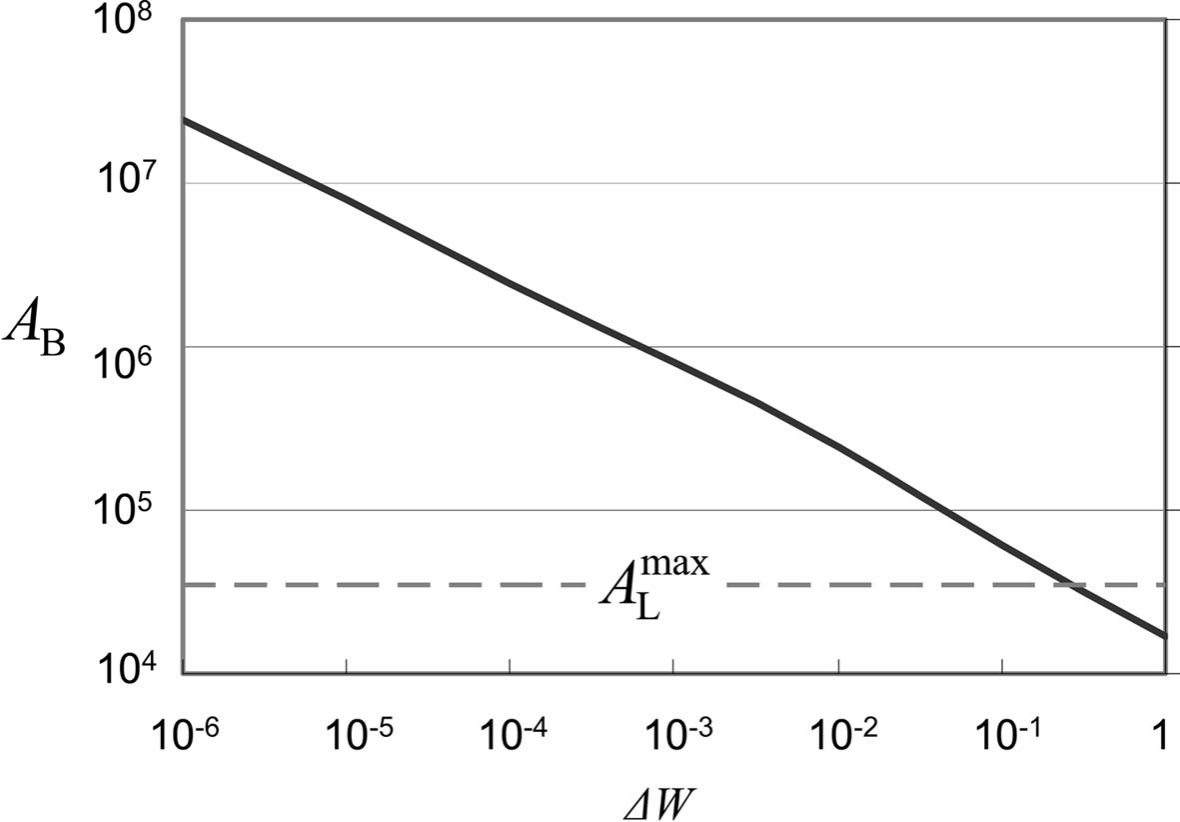
The angular amplification rate (*A*_B_) as a function of Δ*W* for the Si(220) reflection in the Bragg mode. The solid line shows the value of *A*_B_ in equation (21) and the dashed line shows the value of 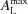.

The angular amplification rate Δγ/Δα, which is defined as the ratio of the divergence angle of the refracted beam Δγ to that of the incident wave Δα, has been analytically derived by Authier (2001 ▸) in the Laue geometry for a non-absorbing crystal. The rate is much larger at the centre (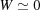) of the reflection region than at the edges (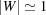). In the symmetric Bragg geometry, the relation between *W* and γ is given by 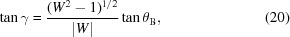
where γ is the angle of the refracted-beam (the Poynting vector) direction from the direction parallel to the surface. The value of γ is 0 for 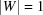 and increases as 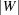 increases. It is close to 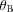 for 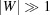. The angular amplification in the symmetric Bragg geometry 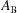 can be written as 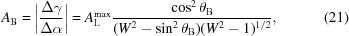
for 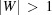. Here 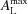 is the maximum angular amplification in the symmetric Laue geometry given by 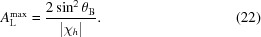
For Si(220), the value of 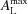 is 3.5 × 10^4^. In the Bragg geometry, 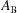 increases as Δ*W* approaches zero, and it is infinite at 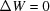, where 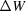 is defined by 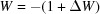. Fig. 10 ▸ shows values of 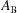 as a function of 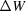. When 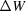 is 10^−3^, 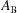 is 7.8 × 10^5^ and 22 times larger than 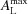. In IFMD for 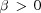, the typical value of 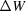 corresponding to the first peak (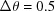) is of the order of 10^−1^. On the other hand, in IFRB for 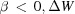 corresponding to the sixth peak (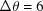) in Fig. 6 ▸(*a*) is of the order of 10^−5^. In order to obtain a high angular amplification rate, it is better to use the interference fringes for 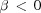.

### 4.3. Coherent condition and energy width of IFRB   

**Figure 11:**
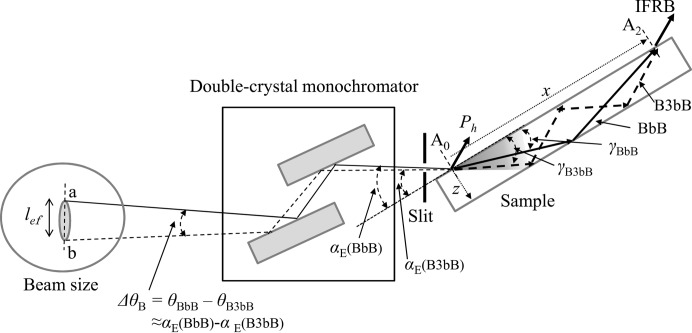
The optical system for evaluating the effective source size and the effective divergent angle for forming IFRB between the beams in the BbB and B3bB modes. By tracing the beam in the BbB mode incident on the sample at *x* with the glancing angle 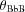 back to the source, it arrives at point *a* in the plane of the source. In a similar way the beam in the B3bB modes arrives at point *b*. The length between the points *a* and *b* gives the effective source size for forming the IFRB at *x*. The effective divergent angle Δθ_B_ is given by 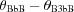.

**Figure 12:**
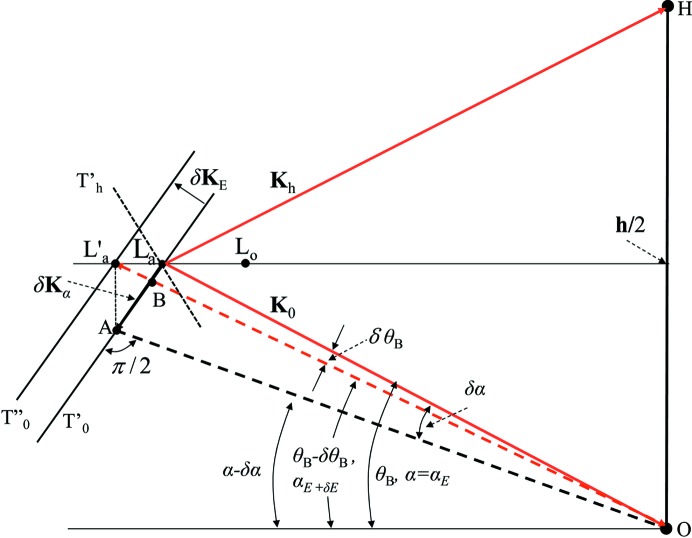
Dispersion surfaces and diffraction geometry in the vacuum. The glancing angle α is taken to be the same as the Bragg angle 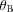, for simplicity. T′_0_ and T′′_0_ represent the dispersion surface for the beam with 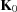 and that with 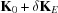, respectively. 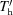 represents the dispersion surface for the beam 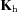. LaO, L′aO and AO represent the vectors 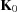, 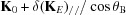 and 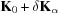, where 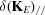 is the component vector of 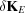 parallel to the lattice plane.

In the present optical system in Fig. 3 ▸(*a*), the divergent angle of the X-rays is estimated to be 670 nrad, as the distance from the source to the slit is 30 m and that from the slit to the sample is 300 mm. The X-ray enters the sample crystal after being reflected from the double-crystal monochromator and passing through the slit. The source size of the X-ray is 60 µm and larger than the width of slit 1 (20 µm). In order to discuss the coherence in this case, the effective coherence length and the source size are evaluated using the optical system in Fig. 11 ▸. At the incident point (A_0_) of the X-ray, the beams in the BbB (solid line) and B3bB modes enter the crystal with the glancing angles 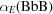 and 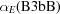. The angle 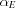 is the glancing angle of incidence for the X-ray with an energy spread of 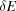 and α is the glancing angle for the monochromatic X-ray. In other words, 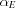 is the angle in the energy-dispersive mode and α is that in the rotating-crystal mode. In the experiment, the incident glancing angle (α) is fixed and the X-ray from the bending magnet is a wave having an energy spread of 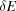 and the corresponding deviation of wavenumber 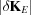. The divergent angle of the X-ray at the incident point on the crystal surface is related to the energy width 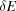, as discussed by Fukamachi *et al.* (2014 ▸, 2015 ▸). The deviations 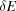 and 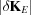 are related to the deviation of the Bragg angle 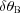 in the crystal as 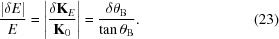
The divergent angle of the incident X-ray corresponds to the deviation of the Bragg angle 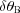. For relating 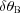 to 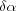, Fig. 12 ▸ shows the dispersion surfaces for the incident wavevectors **K**_0_ and **K**_0_+δ**K** *_E_* with the glancing angle α and the diffraction geometry. For simplicity, the glancing angle α is assumed to be the same as 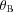. The dispersion surfaces and the diffraction geometry in the more general case are given by Fukamachi *et al.* (2014 ▸, 2015 ▸). The vector 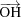 is the reciprocal-lattice vector **h** and **K**_h_ the wavevector of the diffracted wave. The lines T′_0_ and T′′_0_ represent the dispersion surfaces for the wavevectors **K**_0_ and **K**_0_+δ**K** *_E_*, respectively. L_*a*_ and L′_*a*_ are the corresponding Laue points and the distance L_*a*_L′_*a*_ is given by |δ**K** *_E_*|/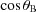. To obtain the same variation of L_*a*_L′_*a*_ by changing the incident angle α (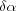), the component of the wavevector 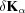 parallel to the lattice plane 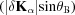 must be L_*a*_L′_*a*_. Then the relations 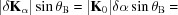 |δ**K** *_E_*|/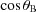 hold. Using these relations and equation (23), 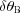 is related to 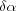 as 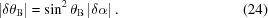
The energy deviation is related to 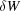 as 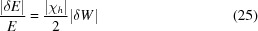
by using the relation 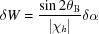
obtained from equation (1) by setting *C* = 1. The effective angle divergence 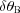 of the incident beam is related to 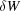 as 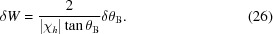
The divergent angle of the beam from a point source corresponds to the deviation of 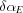 and the deviation of the Bragg angle 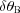 in the present experiment, which gives rise to the deviation of 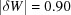 for the Si(220) reflection.

In order to compare the range of 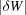 with that needed to excite the refracted beams in the BbB and B3bB modes coherently, the values of 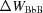 and 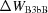 for the beams in the BbB mode and the B3bB mode are determined by using equation (2) and β = −0.02 mm^−1^. Here 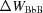 and 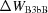 are Δ*W* for the beams in BbB mode and B3bB mode, respectively. The values of 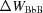 and 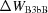 for *x* from 5 to 9 mm are listed in the second and third columns, respectively, in Table 1 ▸. The range of |δ*W*| = 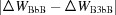 for exciting the IFRB in the range of *x* from 5 to 9 mm is 0.46 and smaller than 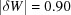. The incident X-ray with the divergent angle of 670 nrad is enough to excite the relevant refracted beams in the BbB and B3bB modes coherently.

Next, the longitudinal coherent condition is discussed. In the present experiment, the values of γ and the path lengths are different for the beams in the BbB and B3bB modes as shown in Fig. 2 ▸(*b*). The value of 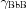 (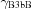) is obtained by putting the value of 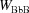 (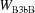) into equation (20). The path length of the beam in the BbB (B3bB) mode is calculated using the values of 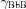 (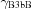) by assuming that the beam trajectory is straight, because the hyperbolic curvature is small. The path length differences Δ*l*_p_ thus calculated from the path lengths of these two beams are listed in the last column of Table 1 ▸. As the longitudinal coherence length 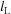 (Born & Wolf, 1970 ▸) is given by 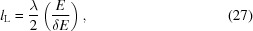
the value is obtained by inserting equation (25) into equation (27) and using 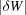. The longitudinal coherence length 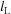 for these beams is listed in the seventh column of Table 1 ▸. At *x* = 8 mm, the path length difference is 58 µm and the coherence length is 204 µm. The coherence length is larger than the path length difference, and the coherence condition is satisfied. On the other hand, the coherence condition is not fully satisfied at *x* = 5 mm, as the coherence length is 51 µm and the path length difference is 93 µm. The interference fringe is blurred around *x* = 5 mm in Fig. 4 ▸(*a*).

The transverse coherence length 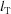 is given by 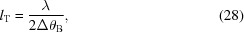
where 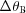 = 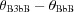 with 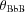 and 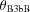 being the Bragg angles for the beams in the BbB and B3bB modes, respectively. The transverse coherence length 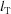 in the present experiment is 186 µm. The effective source size (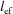) is given by the product of 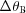 with the distance from the source point to the sample (approximately 30 m). The calculated values of 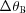 and 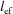 are listed in the fourth and fifth columns, respectively, of Table 1 ▸. As the maximum effective source size is 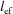 = 9 µm for the formation of IFRB at *x* = 5 mm, the coherence condition is satisfied.

In the observation of IFRB, there is another possible blurring effect caused by the different travelling directions between the beams in the BbB and the B3bB modes. The difference in the directions of these two beams is approximately 300 nrad. As the distance from the sample to the nuclear plate is 100 mm, the resultant difference in the arriving positions on the plate is 30 nm. This difference is much smaller than a period of the IFTB of 0.3 mm and the blurring effect is negligible. A similar blurring effect is expected in the observation of IFMRB and IFTB. This is also negligible in the present setup.

## 5. Summary   

The trajectory of the X-ray refracted beam in a very weakly bent perfect crystal with negative strain gradient shows a hyperbolic form opening down, while it shows a hyperbolic form opening up when the strain gradient is positive. This difference in beam trajectory results in quite different X-ray interference fringes. The interference fringes for β < 0 were mainly studied in the above. In the wave emitted from the entrance surface IFRB were observed between once- and twice-reflected beams from the back surface. In the transmitted wave from the back surface, IFTB were observed between the beam directly reaching the back surface and the beam once reflected from the entrance surface. These interference fringes for β < 0 were compared with the corresponding interference fringes for β > 0. IFRB and IFTB were analysed by using the dynamical theory of diffraction for a bent crystal. The results showed very good agreement between the observed and the calculated values of the interference fringe spacing, which enabled us to evaluate the strain gradient of the sample crystal. If a thinner crystal is used when 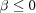, it is possible to observe Bragg–*Pendellösung* fringes reported by Batterman & Hildebrandt (1968 ▸), which are formed by interference between two waves corresponding to the two branches of the dispersion surface as shown by Authier (2008 ▸). But if 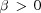, it is not possible to observe them because of the mirage effect.

The fringe spacing of IFRB for β < 0 is smaller than that of IFMRB for β > 0. It is noted that IFRB for β < 0 should be potentially useful for measuring a very weak strain of the order of 10^−7^.

## Figures and Tables

**Table 1:** The deviation parameters Δ*W*_BbB_ and Δ*W*_B3bB_ for the beams in the BbB and the B3bB modes at position *x* from 5 to 9 mm in Fig. 4 ▸(*a*) (in the second and the third columns, respectively) The corresponding effective divergent angle Δθ_B_, source size *l*_ef_, energy width δ*E* and longitudinal coherence length *l*_L_ for formation of the IFRB between these two beams are in the fourth to the seventh columns. Δ*l*_p_ in the last column is the path length difference between these two beams.

*x* (mm)	Δ*W*_BbB_	Δ*W*_B3bB_	Δθ_B_ (nrad)	*l*_ef_ (µm)	δ*E* (meV)	*l*_L_ (µm)	Δ*l*_p_ (µm)
5	0.07	0.47	300	9	10.8	51	93
6	0.04	0.26	166	5	6	92	78
7	0.03	0.17	105	3.1	3.8	146	67
8	0.02	0.12	75	2.3	2.7	204	58
9	0.01	0.09	60	1.8	2.2	255	52
